# Toward passive BCI: asynchronous decoding of neural responses to direction- and angle-specific perturbations during a simulated cockpit scenario

**DOI:** 10.1038/s41598-022-10906-5

**Published:** 2022-04-26

**Authors:** Shayan Jalilpour, Gernot Müller-Putz

**Affiliations:** 1 Institute of Neural Engineering, Graz University of Technology, Stremayrgasse 16/4, 8010 Graz, Austria; 2 BioTechMed, Graz, Austria

**Keywords:** Biomedical engineering, Neuroscience

## Abstract

Neuroimaging studies have provided proof that loss of balance evokes specific neural transient wave complexes in electroencephalography (EEG), called perturbation evoked potentials (PEPs). Online decoding of balance perturbations from ongoing EEG signals can establish the possibility of implementing passive brain-computer interfaces (pBCIs) as a part of aviation/driving assistant systems. In this study, we investigated the feasibility of identifying the existence and expression of perturbations in four different conditions by using EEG signals. Fifteen healthy participants experienced four various postural changes while they sat in a glider cockpit. Sudden perturbations were exposed by a robot connected to a glider and moved to the right and left directions with tilting angles of 5 and 10 degrees. Perturbations occurred in an oddball paradigm in which participants were not aware of the time and expression of the perturbations. We employed a hierarchical approach to separate the perturbation and rest, and then discriminate the expression of perturbations. The performance of the BCI system was evaluated by using classification accuracy and F1 score. Asynchronously, we achieved average accuracies of 89.83 and 73.64% and average F1 scores of 0.93 and 0.60 for binary and multiclass classification, respectively. These results manifest the practicality of pBCI for the detection of balance disturbances in a realistic situation.

## Introduction

Different reasons lead to sudden loss of balance during aviation and driving so that the pilot (we use the term ‘pilot’ generally, as it may include any person steering a vehicle or plane) does not have any control over the vehicle, and it can result, in the worst case, in catastrophic events. For instance, turbulence throws an airplane out of control by imposing irregular motions during windy weather or shear wind. In addition, many reasons, such as human error, mechanical faults, and environmental factors, cause a pilot to feel unstable who may fail to prevent an accident. During these situations, the pilot must make a decision and respond in a very short time to keep own balance and control the vehicle. Maintaining the balance of the vehicle requires a fast and accurate reaction of the pilot to avoid instability, which involves intense mental processing of the pilot and may lead to an incorrect or late reaction if the pilot was not prepared for the situation. The presence of a system that is enabled to take control of vehicles in hazardous moments and compensate for abrupt postural instability faster and more precisely than humans is needed to inhibit harmful consequences of losing balance and, hence, enhance the human–machine interaction (HMI).

With the advancement of neuroimaging techniques, researchers have discovered that balance perturbations are linked to specific neural activities in electroencephalography (EEG)^1–7^. This brain pattern is called perturbation evoked potential (PEP) and has several components containing a small positive potential (P1) and large negative peak (N1)^8–15^. P1 and N1 components are followed by late perturbation-evoked responses (PERs)^16–18^. The amplitude and latency of PEPs are modulated by various factors, such as perturbation velocities and amplitudes^19–29^.

One of the main applications of EEG emerges in brain-computer interface (BCI) systems, where BCIs allow an individual to communicate with the external environment by using brain activity (i.e., EEG) without any movements^30–37^. BCIs are categorized into three types of active, reactive, and passive systems based on the application^38^. An active BCI is adopted by endogenous and conscious brain responses of the user for controlling a system. One obvious example of active BCI is motor imagery tasks, in which the user imagines performing physical actions such as hand or foot movement^39^. In a reactive BCI, the system is controlled when the user intentionally focuses on exogenous stimulations, and the presence of an external stimulus is always needed to modulate the brain signals. P300 spellers are one of the main examples of reactive BCIs, where a user concentrates on visual or auditory stimuli to spell the characters by brain signals^40^. In addition, steady-state visual evoked potentials (SSVEPs) are applicable to reactive BCIs where participants pay attention to a flickering visual target, and brain signals oscillate with the flickering frequency^41^. Finally, the concept of passive BCI has been recently introduced in the BCI field, where the system takes action to perform a command by arbitrary brain activity of the user while the participant does not have voluntary control over the system^42,43^. A pBCI assesses the cognitive state of a person without intentionally involving the user to provide information to a machine or computer. For instance, a neuroadaptive pBCI was successfully used to control the cursor movements in two dimensions without conscious communication of the person^44^. Another example of pBCI is found in the detection of error processing when an unintended response from an external device evokes brain signals and causes a misinterpretation of the interaction between the user and device. An asynchronous pBCI experiment has been studied in spinal cord injury (SCI) and healthy participants to measure this neural pattern known as error-related potential (ErrP)^45^.

In addition, pBCI is able to enrich a human–machine interaction (HMI) by decoding the mental status before the user starts to perform a behavioral action. With respect to the user's balance control, it was shown that EEG can be used to decode PEP from rest EEG in several scenarios^46,47^. Ditz and colleagues investigated whether PEPs are recognizable from ongoing EEG signals when participants sat in a chair, and the chair was tilted manually. They examined the effect of different electrode layouts and window lengths on classification accuracy by using single trials. Ravindran and colleagues employed a grad-class activation map (Grad CAM) based on a convolution neural network (CNN) architecture to predict the balance loss by using EEG signals when participants experienced backwards translations.

In this study, we explored whether it is possible to classify the existence and expression of perturbations from ongoing EEG while participants sat in a glider plane and a robot continuously tilted the glider around the roll axis, keeping the other two fixed. In this experiment, participants received perturbations at random time points in two different directions (left and right) and angles (5° and 10°), and we aimed to examine the influence of different kinds of perturbations on PEPs.

## Materials and methods

### Participants

Fifteen healthy individuals (8 females and 7 males, 20–32 years old) participated in the study. All participants had no neurological disorders and normal or corrected-to-normal vision. All of them received 7.50 euros per hour as monetary compensation. The study was performed in accordance with the Declaration of Helsinki and approved by the local ethics committee of the Medical University of Graz. All participants gave signed informed consent.

### Task and experimental design

**Figure 1:**
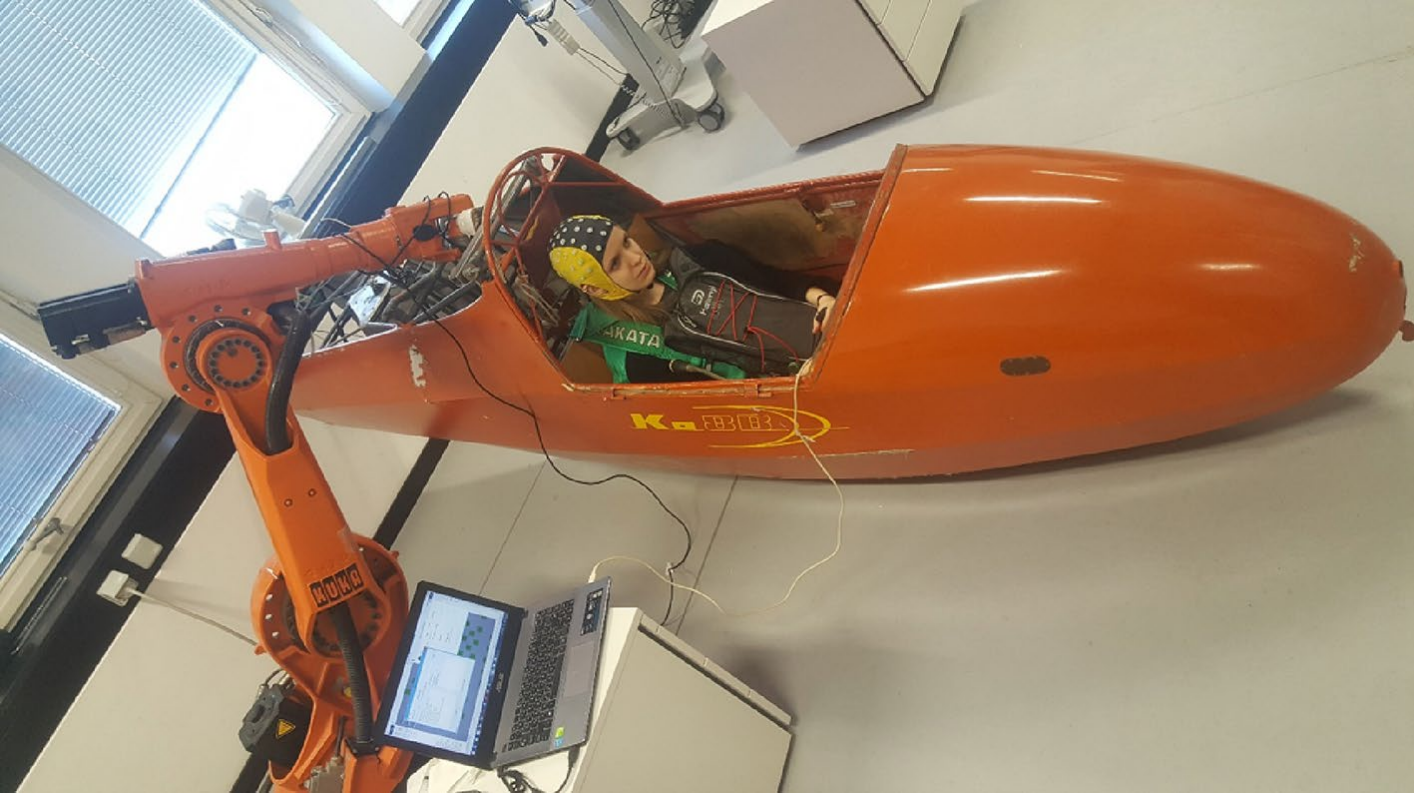
Experimental setup. The KUKA robot was connected to the glider and tilted in the left and right directions at angles of 1.5°, 5° and 10°. The participant fastened a seatbelt to reduce the movements during the experiment. Additionally, the participant kept a bag in front of her in which the EEG amplifier was placed to have the connection with the EEG cap. The participant visible in this figure gave informed consent to publish this figure in an open access publication.

**Figure 2:**
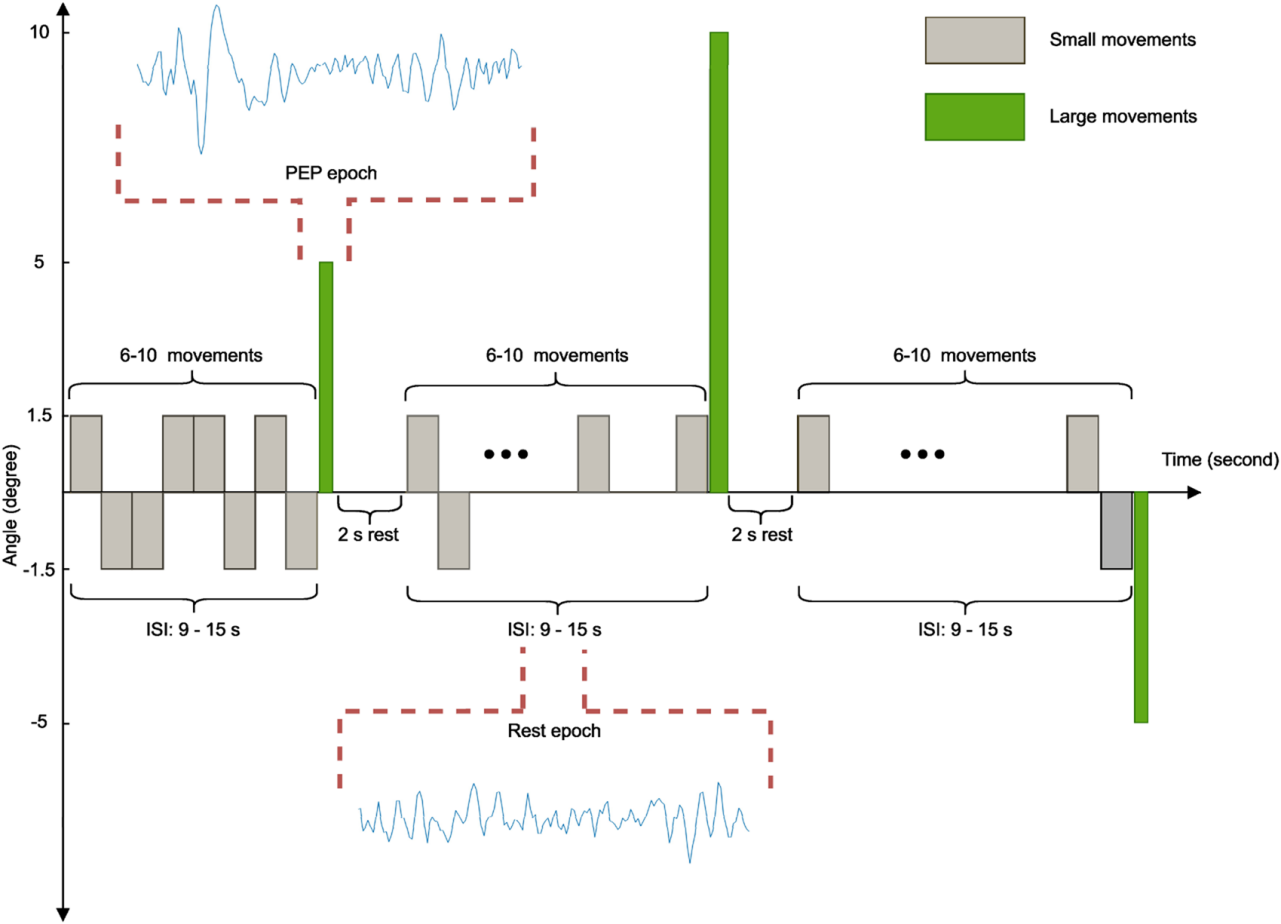
The time sequence of the experiment. The gray bars indicate small movements (1.5 degrees), and green bars—perturbations (5 and 10 degrees), respectively. The interstimulus interval between two consecutive perturbations (large movements) was randomized to [9, 15] s by imposing 6 to 10 small movements. There was a 2-s rest time after the perturbation during that time, the position of the glider did not change, and it stayed at the same position. Additionally, we used the interval between perturbations as rest epochs where small movements were running.

Participants sat in a glider (Ka 8b, Alexander Schleicher GmbH & Co. Segelflugzeugbau) and gazed at a fixation cross at a distance of one meter in front of them. To reduce muscle artifacts and to avoid movements, they were asked to hold the control stick of the glider with both hands, and the stick was fixed in a neutral but immovable position so that it was not possible to move (Fig. 1). A KUKA KRC1 robot (Kuka AG, Augsburg, Germany) was programmed to move the glider in two different directions (left and right) with three tilting angles of 1.5°, 5°, and 10°. We designed an oddball paradigm^48,49^ to make the perturbations unpredictable for participants in a way that the participants experienced two kinds of movements consisting of small (standard stimuli) and large movements (target stimuli). Small movements took place at low speed and more often than large movements, while participants rarely underwent large movements (perturbations) at high speed during the experiment. For this purpose, 6 to 10 small movements were considered between consecutive perturbations, which resulted in an interstimulus interval (ISI) of 9–15 s. Standard stimuli occurred in both directions with an angle of 1.5°, and perturbations occurred in both directions with tilting angles of 5° and 10°. These rare perturbations were perceived as a threatening event for participants, and they resulted in the appearance of perturbation-evoked potential. The time scheme of the paradigm is demonstrated in Fig. 2, illustrating the timing of small and large movements. The robot continuously tilted the glider in the left and right directions during the small movements until a perturbation occurred. Then, the glider stayed at a tilted position for 2 s and returned to the balance position at low speed.

The participants performed 6 blocks in the experiment in which each block included 40 perturbations (large movements) of two different angles (5° and 10°) and directions (left and right). Forty trials consisted of 10 repetitions of each direction and angle. All trials were exposed in a random order, and a maximum of 2 successive perturbations occurred for an identical angle and direction. A video of the experiment can be found in the Supplementary Material.

### EEG data collection

EEG signals were recorded at a sampling rate of 512 Hz using an EEGO amplifier (ANT-neuro, Enschede, Netherlands). Sixty-three active shielded Ag/AgCl electrodes were used with reference and ground electrodes located at CPz and AFz, respectively. The electrode positions were according to the International 10–5 system and were located over frontal, central, parietal and occipital areas. The electrode layout is displayed in Fig. 1 of Supplementary Material. EEG data analyses were performed using MATLAB 2019b (The MathWorks), and we utilized the EEGLAB toolbox^50^ and Berlin Brain-Computer Interface (BBCI) toolbox^51^ for electrophysiological analysis.

A Myo armband (Thalmic Labs Inc, Kitchener, Canada) was used to detect the movement onset by measuring the acceleration simultaneously with EEG data. The armband was mounted on the subject’s right arm, and acceleration data were sampled at 50 Hz. EEG signals and accelerator data were synchronized using the lab streaming layer (LSL) protocol^52^.

### EEG data preprocessing

**Figure 3:**
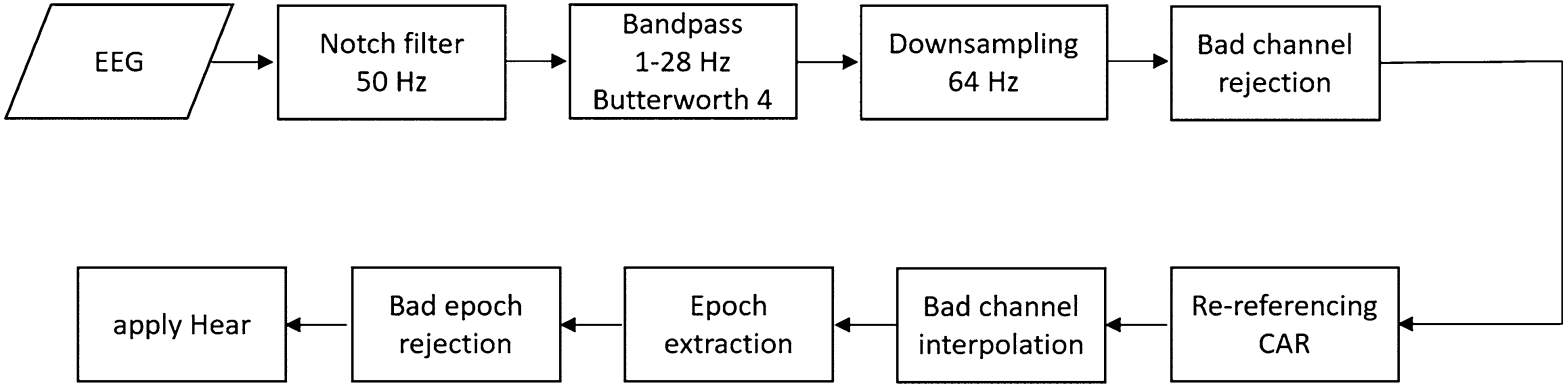
EEG preprocessing pipeline. Brain signals were band-stop (notch) and bandpass filtered, and bad channels were removed after downsampling. Then, data were re-referenced, followed by specific interpolation of removed channels. Next, noisy trials were rejected after extracting the epochs. The pipeline was the same for the online part, except for the 'Bad epoch rejection' block.

The preprocessing pipeline is depicted in Fig. 3. First, power line noise was removed from the data by a notch filter at 50 Hz. Then, we used a causal Butterworth filter of order 4 to filter the data between [1, 28] Hz. The filtered data were downsampled to 64 Hz to fasten the computational process, and after that, bad channels were identified and removed if they correlated with neighborhood channels lower than 75%^53^. We then applied common average reference (CAR) to rereference the data and interpolated the noisy channels. In the next step, the onset of perturbations was obtained by thresholding the acceleration data recorded from the Myo armband. We segmented the data to [−0.06, 1] s epoch after the onset of each perturbation, and the remaining trials were acquired by using the time interval [−7.06, −6] s before the perturbation onset as rest trials. To exclude noisy trials, we rejected contaminated trials based on three different statistical parameters^54^ (amplitude threshold of ± 150 µV, abnormal joint probability, and kurtosis with a threshold of 4 STD). On average, we rejected 19% of the trials as noisy trials meaning that 129 out of 160 perturbation trials were used for classification. Finally, we exploited the high-variance electrode artifact removal (HEAR) method^55^ to reduce channel pops and drifts in the signal. The same preprocessing pipeline was used for both offline and online conditions, apart from the bad epoch rejection part, which was applied on offline blocks only.

### Asynchronous PEP detection

We split the data into 3 partitions to simulate an online scenario. The first partition, consisting of four blocks (160 PEP and 160 rest epochs, excluding rejected trials), was assigned as calibration data to build the classifiers and obtain parameters in offline mode (calibration training). The second partition (5th block) was used to tune the classifiers in asynchronous mode (asynchronous modeling), and the third partition (6th block) served as unseen data and was considered for validation of the asynchronous model (asynchronous validation).

A hierarchical structure was designed to continuously detect the existence and expression of perturbations. First, we applied a binary classifier to determine whether perturbation appeared. If the first classifier confirmed PEP detection, then 4-class classification was performed to identify the expression of perturbation.

### Feature extraction and classification

#### Binary classifier

To incorporate the spatial and temporal characteristics of the PEP, we used the BCSP^56^ (bilinear common spatial pattern) method for extracting the features in binary classification. This method finds the pairs of filters (e.g., spatial and temporal filters) that multiply in two directions of the matrix EEG data (channels × times) in order to maximize the power ratio between the two different classes. Calibration data, including the perturbation and rest trials, were used to extract the CSP filters. The optimal number of spatial and temporal filters was determined by performing 10 times tenfold cross-validation on calibration data. Then, the obtained filters were multiplied by calibration and online data, and diagonal elements of spatial and temporal covariance matrices were used as features^57^.

#### Multiclass classifier

In the 4-class scenario, we took the amplitudes of all 63 EEG channels at each time point and selected the most important features by exploiting the Fisher algorithm. The best features were chosen by applying 10 times tenfold cross-validation on calibration data.

Additionally, we defined a *time-lag* parameter to find the average detection time of perturbations, and used this parameter to train direction- and angle-specific classifier. This parameter corresponds to the time difference between the perturbation onset and classifier detection time. We compensated for this time difference in a multi classification scenario by shifting the epoch interval. We calculated the *time lag* for each trial of the 5th block, then averaged the time lags over all trials. Finally, we trained a multiclass classifier by incorporating the lag for each specific participant, and validated the model on the 6th block.

In both binary and multiclass classification, features were normalized to have zero mean and standard deviation of one, and radial basis function support vector machine (RBF-SVM) was used to train the classifiers.

### Online classification

#### Asynchronous modeling

In the asynchronous manner, we continuously classified the 5th block using a sliding window of 1.06 s width, with a step of 15.625 ms. The probability of a binary classifier determines whether each window belongs to either class (PEP or rest). The F1 score was considered for evaluating PEP detection in the online mode by integrating true positive (TP), false positive (FP), and false negative (FN) information. In the binary asynchronous classification, the number of true negatives (rest epochs) is much higher than the number of true positives (PEP epochs), and the F1 score is the best metric to relinquish the true negatives in assessing the performance of classifiers. This metric is defined as a weighted average of precision and recall, with a score between 1 (perfect precision and recall) and 0 (worst score). The F1 score assesses a model based on a balance between precision and recall. The optimal threshold regarding the classifier's probability was pinpointed to maximize the F1 score by testing 500 thresholds.

$${\text{Precision }} = \, \left( {{\text{TP}}} \right)/\left( {{\text{TP}} + {\text{FP}}} \right)$$

$${\text{Recall }} = \, \left( {{\text{TP}}} \right)/\left( {{\text{TP}} + {\text{FN}}} \right)$$

$${\text{F1 }} = { 2} \times \left( {{\text{Precision}} \times {\text{Recall}}} \right)/\left( {{\text{Precision}} + {\text{Recall}}} \right) \, = \, \left( {{2} \times {\text{TP}}} \right)/\left( {{2} \times {\text{TP}} + {\text{FP}} + {\text{FN}}} \right)$$

The classification procedure was performed as follows: if the classifier detected a perturbation, then the sliding windows until two seconds after that would be ignored for classification as a refractory period. The detected trial went through the second classifier to recognize the expression of perturbation.

#### Asynchronous validation

In the asynchronous validation part, we evaluated the asynchronous model by using the subject-specific time lag and probability threshold for PEP classification. The F1 score and classification accuracy were considered to assess the binary and multi-classification results. The accuracy determines the percentage of perturbations which were detected correctly. In order to have a metric to assess the PEP detection in the online scenario, we computed the F1 score which can deal with unbalanced data by incorporating FN and FP.

## Results

### PEPs electrophysiology

**Figure 4:**
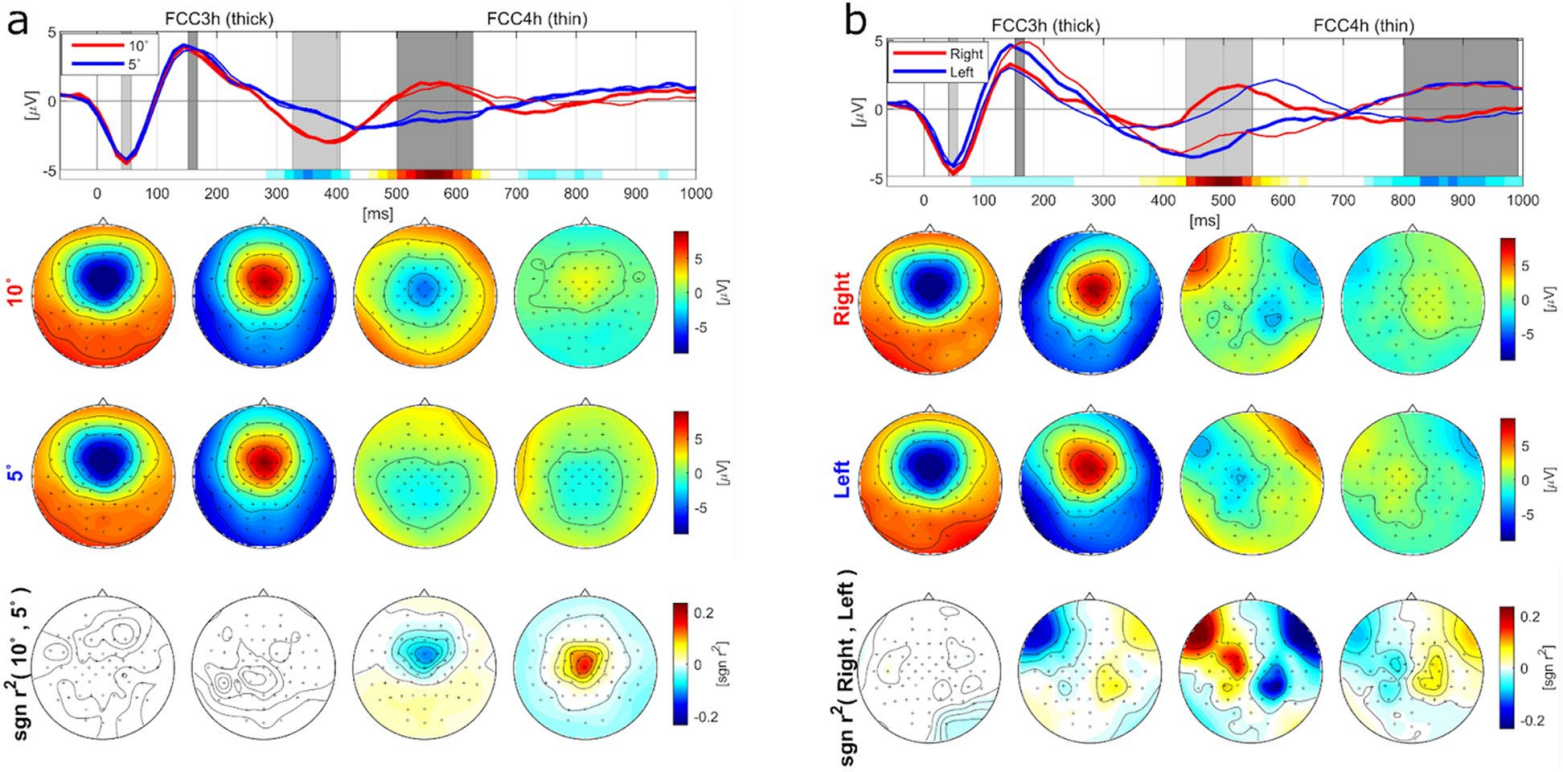
Grand average PEPs over all the participants on the offline data, time-locked to the perturbation onset obtained from the accelerometer. The first row shows the grand average EEG plots for FCC3h and FCC4h in thick and thin lines, respectively. The second and third rows represent the scalp for each condition, and the last row indicates the signed r2 between two classes. The scalp topographies refer to the shadowed areas in the first row plot. (**a**) Grand average potentials and scalp plots are depicted for 10 (red) and 5 (blue) degree perturbations. (**b**) Grand average potentials and scalp plots are represented for right (red) and left (blue) perturbations.

Figure 4 represents the grand average EEG potentials of the calibration blocks at channels FCC3 h and FCC4 h (thick and thin lines, respectively). The standard deviation of PEPs and accelerometer data were plotted in Supplementary Material. Figure 4a shows the grand averaged PEP for 5- and 10-degree classes in the top row, and the red and blue lines depict the brain responses to 5- and 10-degree perturbations, respectively. Topographical maps underneath illustrate the mean amplitudes in the shaded time intervals of PEP plots. The scalp map in the last row depicts the dispensation of the signed r2 coefficient between two classes. As it can be seen, N1 and P2 components have similar patterns in both 5- and 10-degree perturbations, and the r2 coefficients are close to zero in the time course of the N1 and P1 potentials. In the third and fourth time intervals, negative and positive brain activity were observed in the central regions, respectively. The prominent difference between the two classes is evident in the period of 320–620 ms after the perturbation onset.

The grand average PEPs for right and left perturbations with the corresponding topographies are plotted in Fig. 4b. Similar to Fig. 4a, four shaded periods indicate the scalp topographies of EEG potentials. The N1 component is specified in the first time interval, and it looks the same in right and left perturbations. In the second, third, and fourth time intervals, distinct brain responses are noticeable between the two classes. The P2 component at approximately 150 ms is mainly focused on the right hemisphere for right perturbations, while for left perturbations, P2 is distributed in the left hemisphere. In the time interval between 420 and 560 ms, different brain activities appear in frontocentral areas.

### Statistical analysis

A one-sided Wilcoxon rank-sum test was applied to calibration data to find significant differences between different angles and directions. The nonparametric test was performed on an average of four designated time intervals and each channel separately. We adjusted the *p*-values for multiple comparisons (n = 252 comparisons) by using the Bonferroni correction.

**Figure 5:**
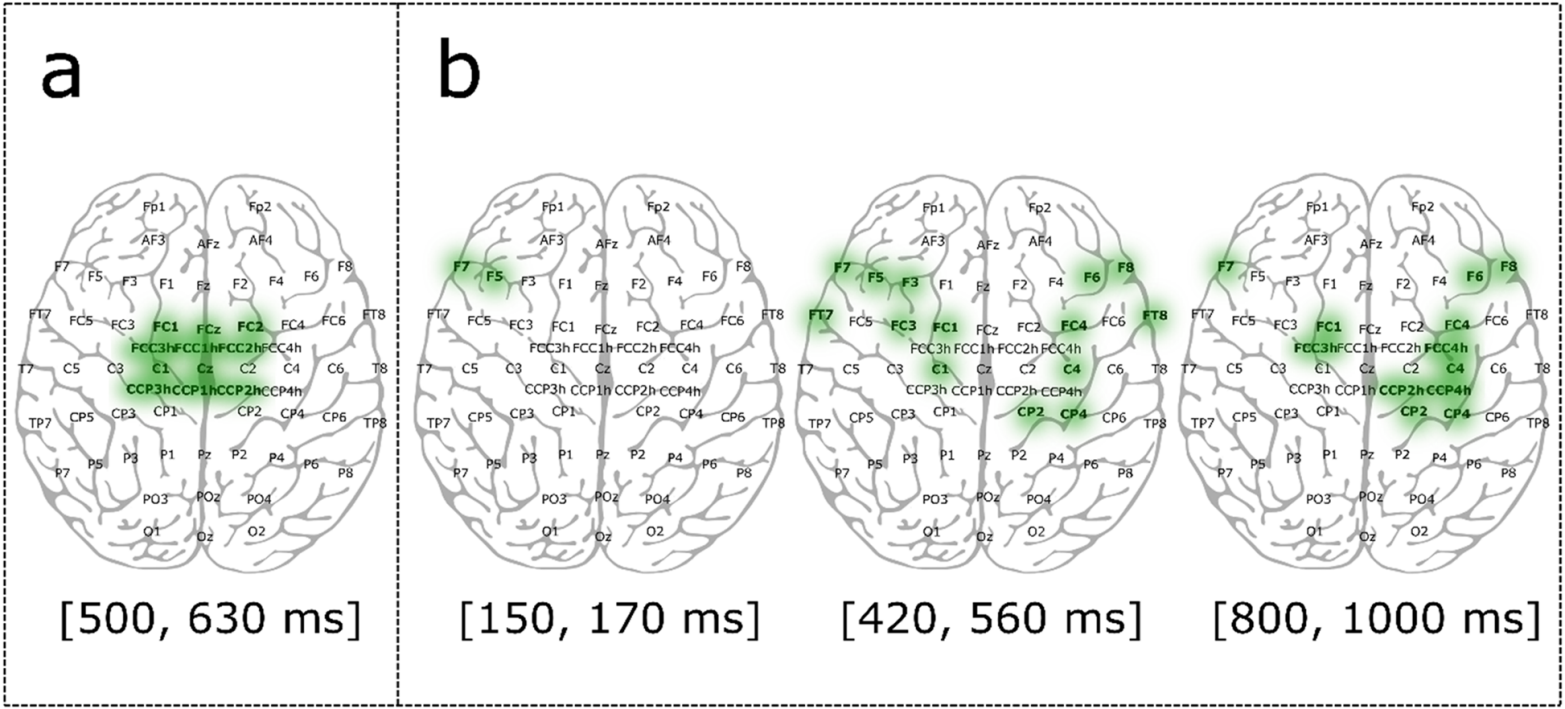
Statistical results obtained by applying the Wilcoxon test, α < 0.05, with Bonferroni correction for n = 252 comparisons. (**a**) Significantly different electrodes between 10 and 5 degree perturbations are marked in the central region in the time period of [500, 630] ms. (**b**) Significantly different electrodes between right and left perturbations are shown in three time intervals of [150, 170], [420, 560] and [800, 1000] ms.

Statistical analysis revealed no significant differences between different angles in the N1 and P2 components. Furthermore, no differences were found in the third interval between 5- and 10-degree trials. The fourth time interval in Fig. 4a revealed a significant difference between 5- and 10-degree perturbations in central areas. We marked the significant electrodes acquired from the test in Fig. 5a. It is apparent in Wilcoxon test results that diverse brain activities were centralized in the center part of the scalp.

Similarly, the N1 component appeared for right and left perturbations, while the P2 component showed meaningful differences in the F7 and F5 channels. Additionally, right and left perturbations differed statistically in the third and fourth time intervals of Fig. 4b. Figure 5b depicts the brain regions that resulted in significant differences. Distinct late perturbation-evoked responses (PERs) were lateralized in frontocentral electrodes between right and left perturbations.

### Classification

**Figure 6:**
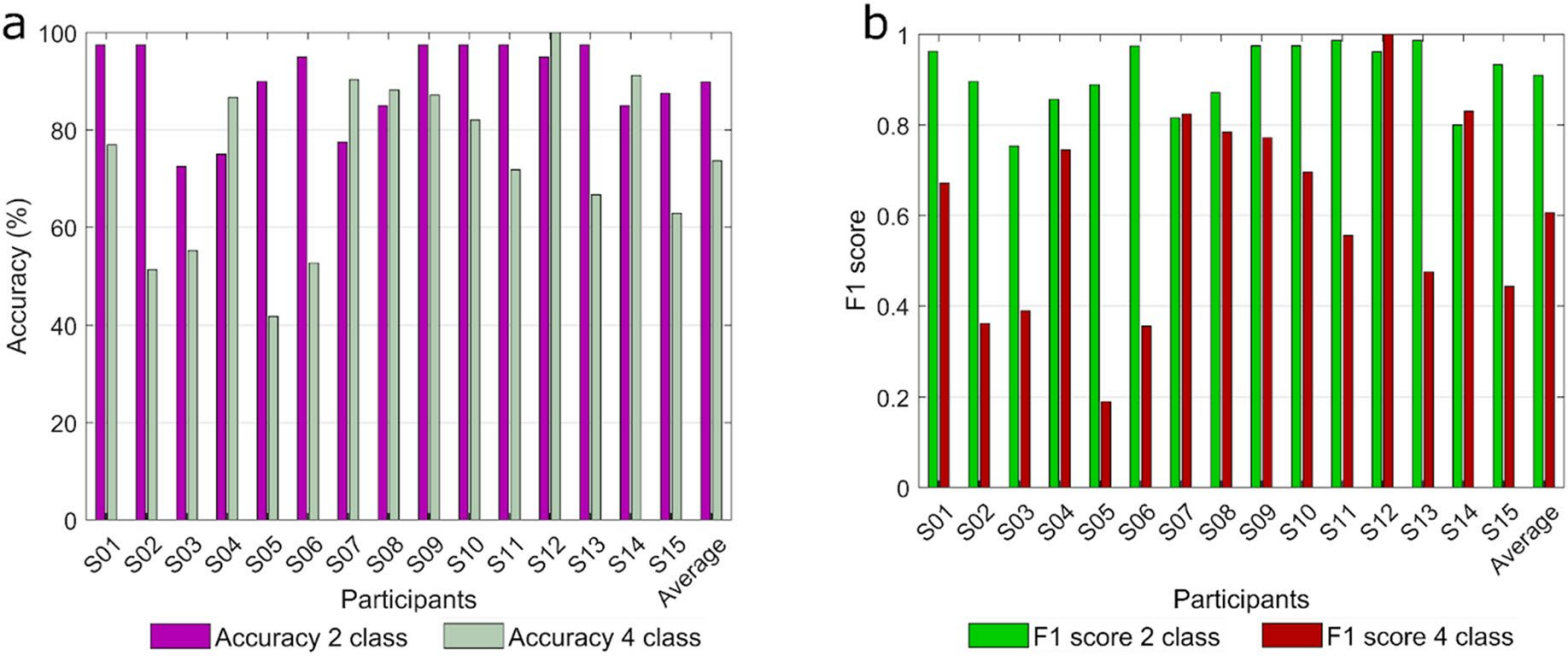
Online asynchronous classification accuracy and F1 score of the hierarchical approach per subject. (**a**) Classification accuracy of each participant is shown as a percentage. The magenta and gray bars represent the binary and multiclass performances, respectively. (**b**) F1 scores of each participant are displayed in green and red colors with regard to the two and four classes.

**Figure 7:**
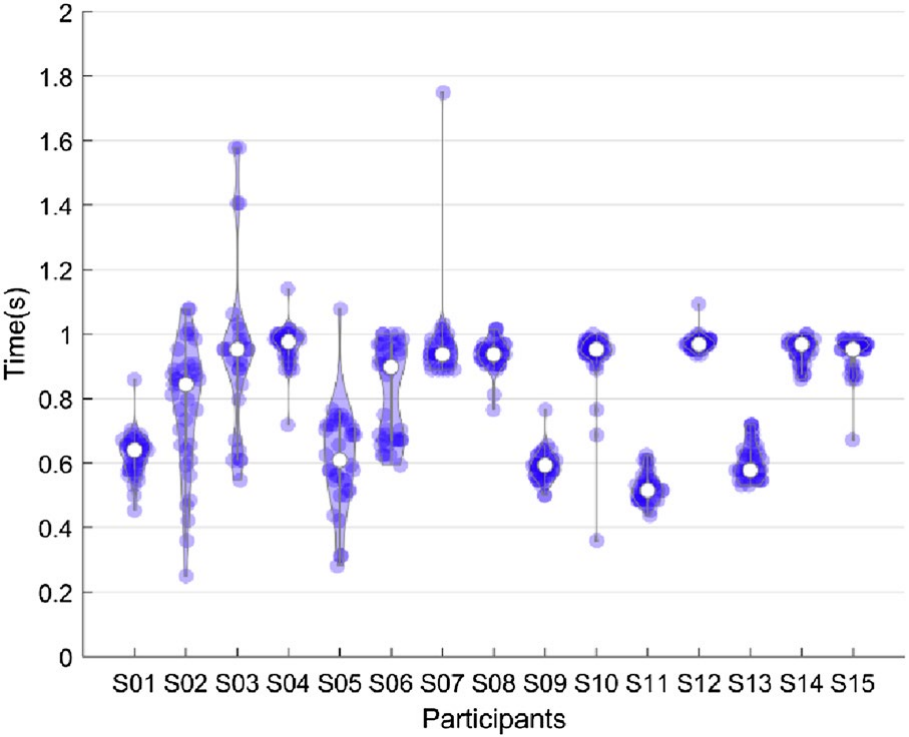
PEP detection time in asynchronous validation. The time points were distributed between 0.2 and 2 s in relation to perturbation onset. The average detection time of all subjects was less than 1 s.

Figure 6 displays the achieved results on asynchronous data using the hierarchical model. The F1 score and classification accuracy are considered evaluation metrics in the online simulation. Additionally, the detection time (*time lag)* of the binary classifier is provided in Fig. 7 to provide better insights into the results. Average accuracies of 89.83% and 73.64% were gained for binary and multiclass classification. All of the participants could yield an accuracy higher than 70% in binary classification (Fig. 6a), showing promising results in an asynchronous experiment.

Figure 6b presents the achieved F1 score of every participant as well as the average of all participants for binary and multiclassification. False positive (FP) trials are defined as an error in classification in which the classifier detected perturbation incorrectly while no perturbation was present. It means that FP happens when no perturbation occurred but the classifier detected a perturbation. Additionally, false negative (FN) trials indicate the condition where the classifier failed to detect perturbation when perturbation occurred. In other words, FN is expressed when a perturbation occurred but the classifier couldn’t recognize it. Finally, we defined true positive (TP) trials where the classifier correctly detected perturbation. In the four classes scenario, we calculated the F1 score per class in a one-vs-rest manner, and then took the mean of each class’s F−1 score by using a macro averaging method. Also, we just considered the trials in which perturbation was detected correctly in the binary classification. We obtained average F1 scores of 0.93 and 0.60 for binary and multiclass scenarios, respectively. Moreover, we computed the true positive rate (TPR) for binary and multiclass classification, and presented the TPR of each participant in Fig. 5 of Supplementary Material.

Figure 7 displays the time course of PEP detection for every participant within the trials with respect to the perturbation onset (t = 0 s). An average detection time of 800 ± 200 ms (mean ± std) was acquired in asynchronous validation. As shown in Fig. 7, subjects 2, 3, 5, and 6 had spread distributions, meaning that they had diverse detection times, and they reached the lowest accuracies in multi-classification (Fig. 6a).

**Figure 8:**
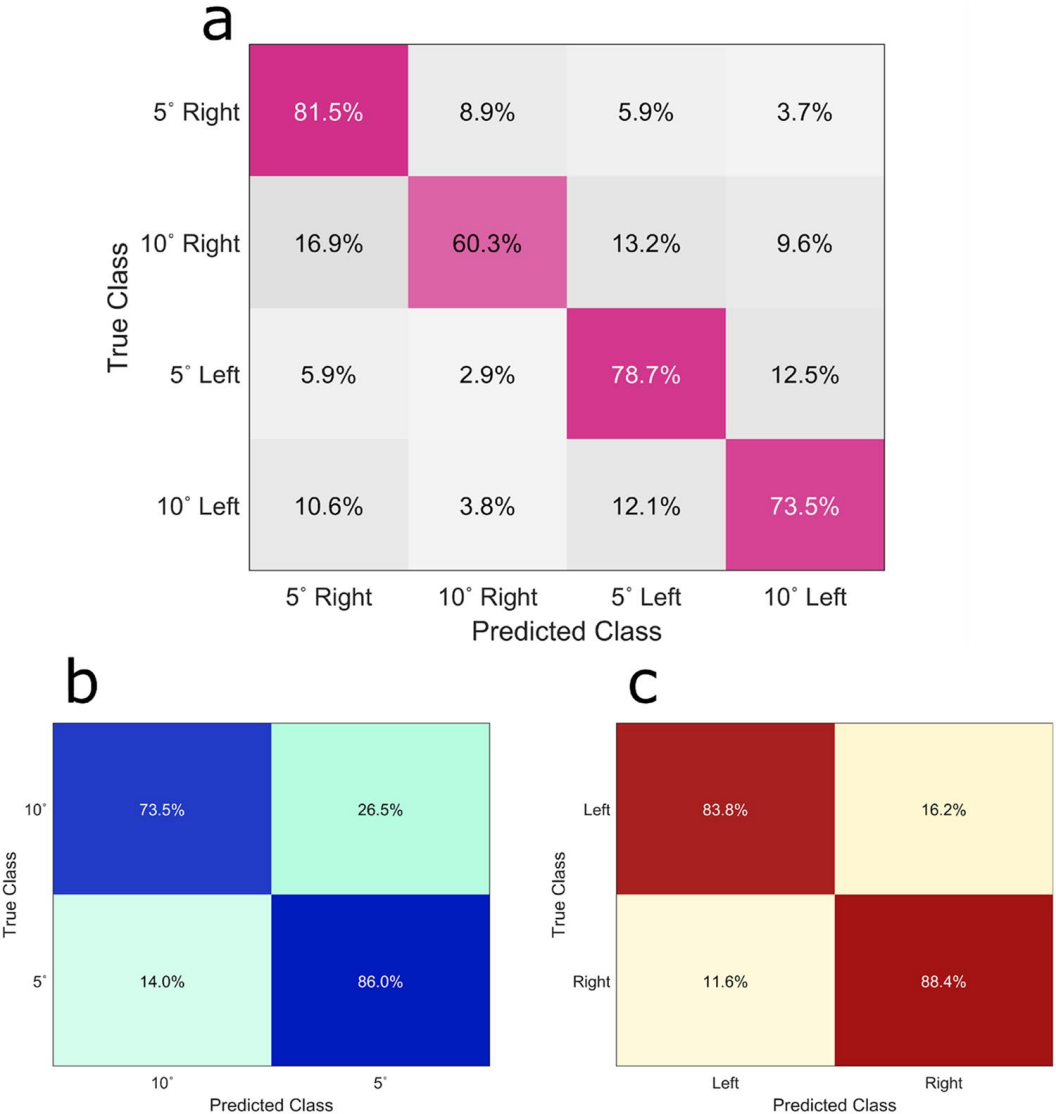
Confusion matrices of perturbation expressions. (**a**) The confusion matrix of 4 class classifications was plotted for four conditions. (**b**) The confusion matrix of angle-specific perturbations. (**c**) The confusion matrix of direction-specific perturbations.

Figure 8 depicts the confusion matrices in the asynchronous phase in a multiclass manner. Figure 8a shows that we could successfully separate the four classes with promising accuracy. In Fig. 8b and c, we investigated the performance of the model to discriminate the angles and direction. Figure 8b shows the classification accuracy of two angles without considering the direction of movements. We could distinguish the 5- and 10-degree perturbations with average accuracies of 73.5 and 86%, respectively. The confusion matrix in Fig. 8c indicates the obtained accuracy for each direction without considering the angle of perturbation. Left and right trials were detected with accuracies of 83.8 and 88.4%, respectively, and an average accuracy of 86.09% was obtained for direction classification.

## Discussion

In the described study, we detected and analyzed perturbation evoked potentials in a simulated cockpit experiment. Our findings indicated that the expression of perturbations could be classified reliably in single trials. We generated the perturbations by abruptly moving the glider in two different directions and angles. The perturbations had different acceleration for two angles, and the accelerations were set to cause a sudden movement so that it was surprising enough to evoked PEPs. Furthermore, speed and acceleration of small movements were selected in a way that they were slow enough to not throw participants of balance.

Furthermore, we evaluated the feasibility of PEP detection in a simulated online manner, demonstrating the utility of this study in realistic applications. To the best of our knowledge, this is the first study that explores PEP detection with different expressions of perturbation in an asynchronous scenario. Our results showed no differences between N1 components induced during four expressions of perturbations, and it had a similar pattern around frontal and central regions in all conditions. The P2 component was spread in frontocentral areas with a maximal peak between 120 and 200 ms after perturbation onset. We observed no distinction in P2 components between different tilting angles, but slight differences were discovered between the right and left directions. Previously reported studies^2,47^ found no differences between different directions of perturbation in the N1 and P2 components. Dietz and his colleagues^2^ imposed stance perturbation in forward and backward directions, while they recorded three channels including C3, Cz and C4. Also, Ditz and colleagues ^47^ found differences in P2 components before applying referencing (when the left mastoid was used as a reference channel), but no differences were obtained after EEG channels were referenced by the CAR algorithm. Our results proved that the P2 component was laterized in the right hemisphere for right perturbations and vice versa, which is in conflict with previous studies and could be described by different EEG setups and analyzing procedures. Additionally, it was shown that the forward and backward perturbations can be decoded by using the spectral information (3–10 Hz) of brain signals in the frontocentral areas^58^. Our findings provide evidence that direction-specific instability was encoded across the fronto-centro-parietal region, which is in accordance with the reported study.

Another important finding in our study is that different neural activities are discernable from the EEG signals for 5- and 10-degree perturbations. Although 5- and 10-degree trials had the same stimulus characteristics in terms of perturbation amplitude, 10-degree perturbations had longer durations than 5-degree perturbations. Based on the achieved results, we discovered that the central cortical rhythms in the time range of 500 and 650 ms after perturbation onset play a significant role in discerning the 5- and 10-degree perturbations.

Furthermore, the average classification accuracies show that directions can be differentiated with higher accuracy than angles of perturbations. This conclusion is aligned with the obtained results in PEP electrophysiology, where signed r2 coefficients of different directions exhibited more differences in comparison with different angles. Also, our analysis revealed that PEPs could be distinguished with an average of 800 ms. This feasibility is a vital requirement in human–machine interfaces where we want to control the balance in an online and realistic situation. Therefore, detecting an imbalance situation and compensating it should be done in the fastest way, and it depends how quick the BCI system can recognize the perturbation. We showed that all the participants had average detection time less than 1 s after the perturbation which is promising results to make use of pBCI for vehicular or rehabilitation contexts in the future.

We implemented an online simulation in this study that exploited a hierarchical approach to distinguish perturbations from ongoing resting EEG and then decoded the expression of perturbation. We assessed the performance of the classifiers with regard to classification accuracy and F1 score. We reached an average accuracy of 89.83% in the perturbation detection scenario and 73.64% in the 4-class scenario. We did not compare the system’s performance with a chance level since the number of trials should be taken into account for calculating the chance accuracy^59^, and it is not reasonable to exploit the chance accuracy in an online asynchronous classification where we have many epochs with unbalanced classes. In the asynchronous condition, all participants exhibited average PEP detection within one second after perturbation onset. Various detection times of the participants can be associated with different perturbation onset, which was measured by acceleration data.

In conclusion, we were able to robustly classify the direction and angle of perturbations from ongoing EEG signals in an online simulation. The results showed that the average accuracy of binary classification (89.83%) was higher than the average accuracy of four-class classification (73.64%). This drop in accuracy could be associated with a high variance in perturbation detection time. Ditz and colleagues^47^ achieved an average accuracy of 87.6% between PEPs and rest EEG in the offline scenario, while we could reach a sligthly higher, but comparable, accuracy (89.83%) in the binary classification (PEPs vs. rest EEG) in an simulated online study. As part of future work, we will develop analysis methods to identify perturbations with more accurate timing and less variance. Furthermore, we tried to simulate a cockpit scenario similar to a real flying task, although the participants did not perform any specific task during the experiment except gazing at a fixation cross, which makes the study far from realistic. To overcome this limitation, we will conduct future research in real-world scenarios where participants are involved in demanding tasks by implementing flying/driver simulators and virtual reality (VR). Our findings provide the basis for the development of a passive brain-computer interface (pBCI), which can be used as an assistive technology for compensation of sudden deviations during realistic flying/driving.

## Supplementary Information

Supplementary Video 1.

Supplementary Information 1.

## Data Availability

The data provided in this study are available upon reasonable request from the corresponding author.
