# Supplementary material for: Toward passive BCI: asynchronous decoding of neural responses to direction- and angle-specific perturbations during a simulated cockpit scenario
Source: Sci Rep. 2022 Apr 26;12:6802. doi: 10.1038/s41598-022-10906-5 (PMC9042920; doi:10.1038/s41598-022-10906-5)
Supplement: Supplementary file 2 — Supplementary Information 1. [file 41598_2022_10906_MOESM2_ESM.docx]

**Supplementary: Toward passive BCI: Asynchronous decoding of neural responses to direction- and angle-specific perturbations during a simulated cockpit scenario**

Figure 1 indicates the position of EEG electrodes according to the international 10–5 system.


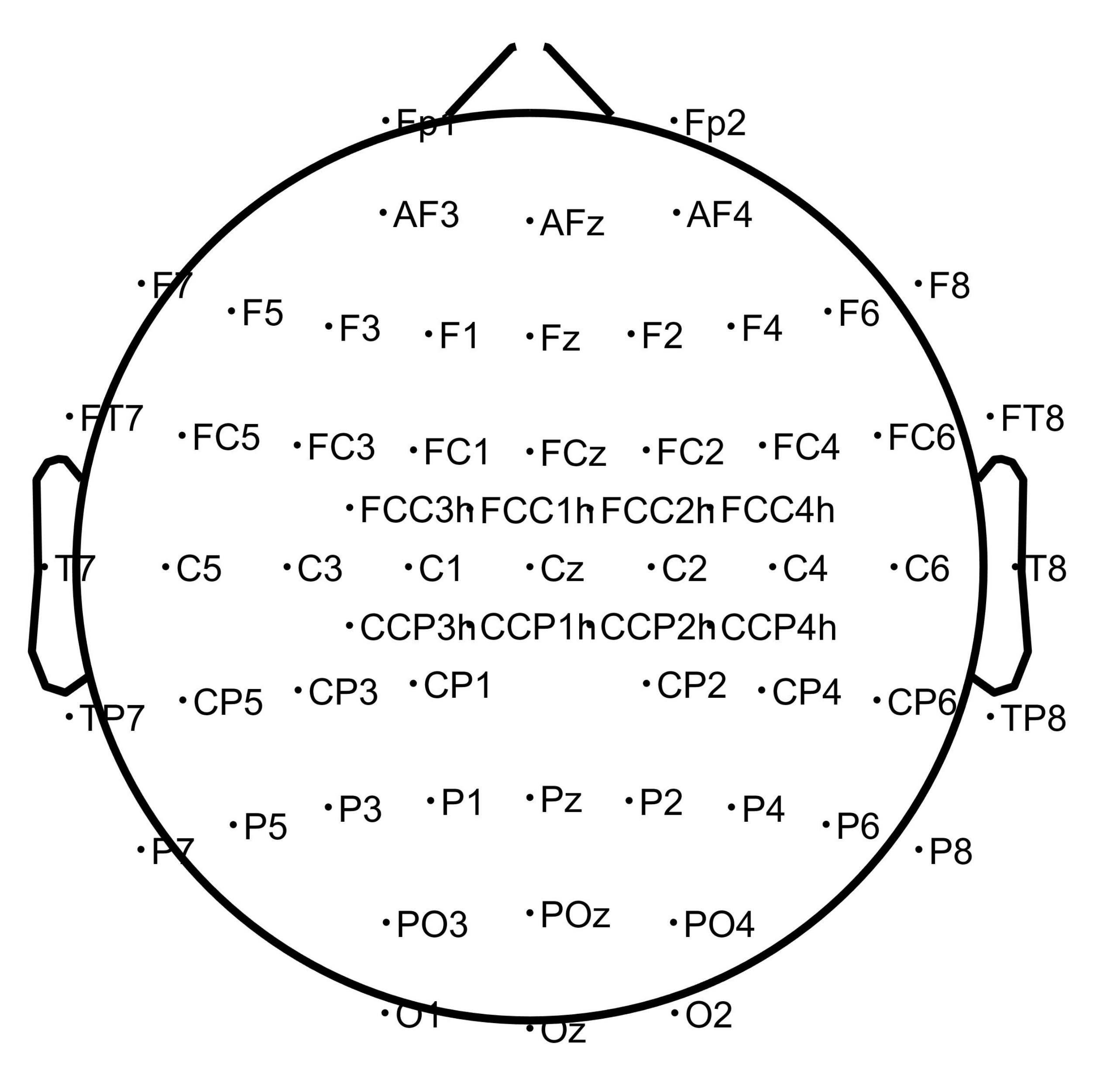


Figure 1. The location of the 63 EEG electrodes. All channels were referenced to CPz channel with a forehead (FPz) ground.

The corresponding plot including the mean and STD of PEPs were shown in Figure 2 for right and left trials. In addition, Figure 3 depicts the brain response of 5 and 10-degree trials.


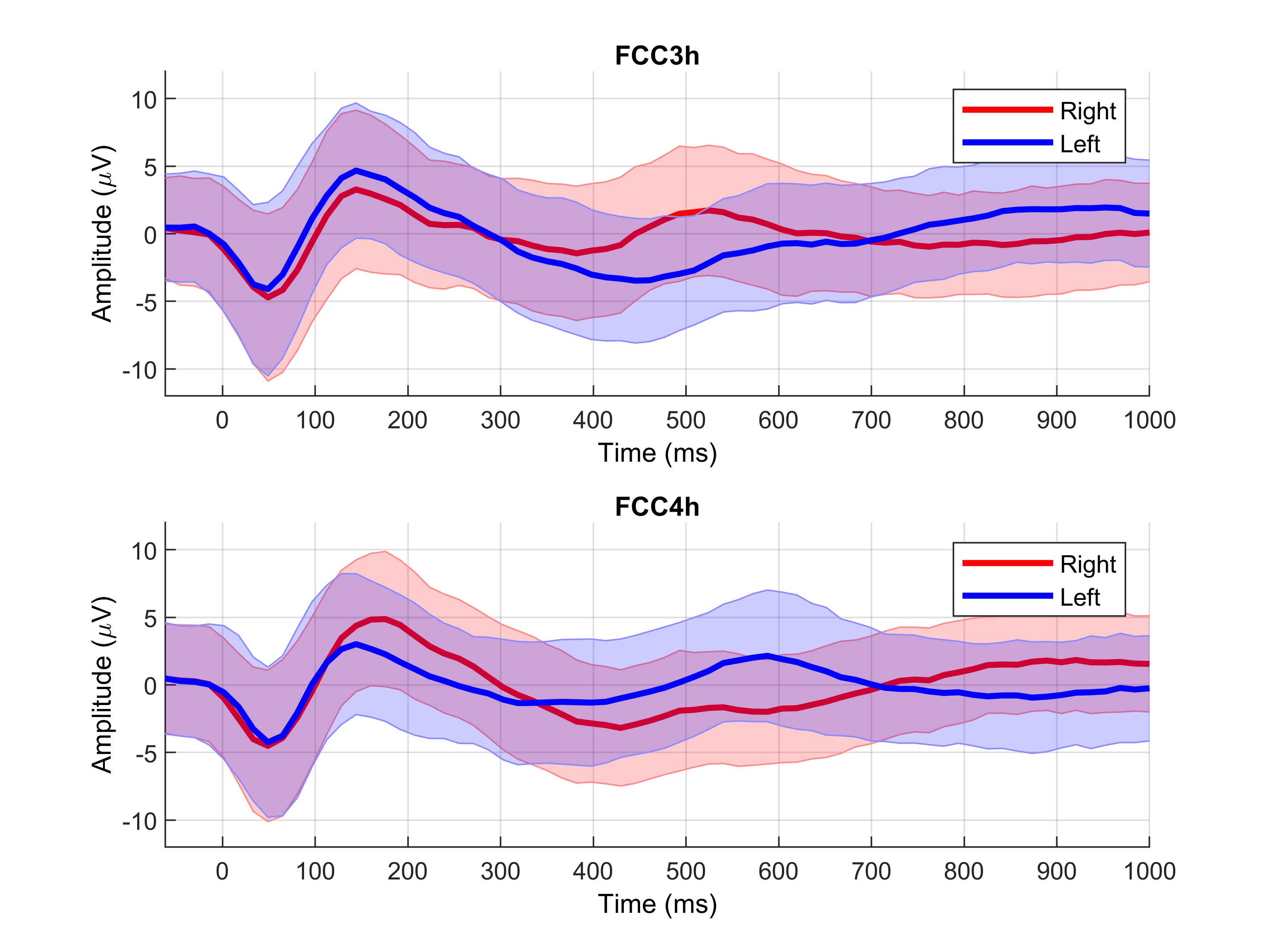


Figure 2. The first and second rows represents the PEPs of FCC3h and FCC4h respectively. Red and blue lines indicate the right and left trials


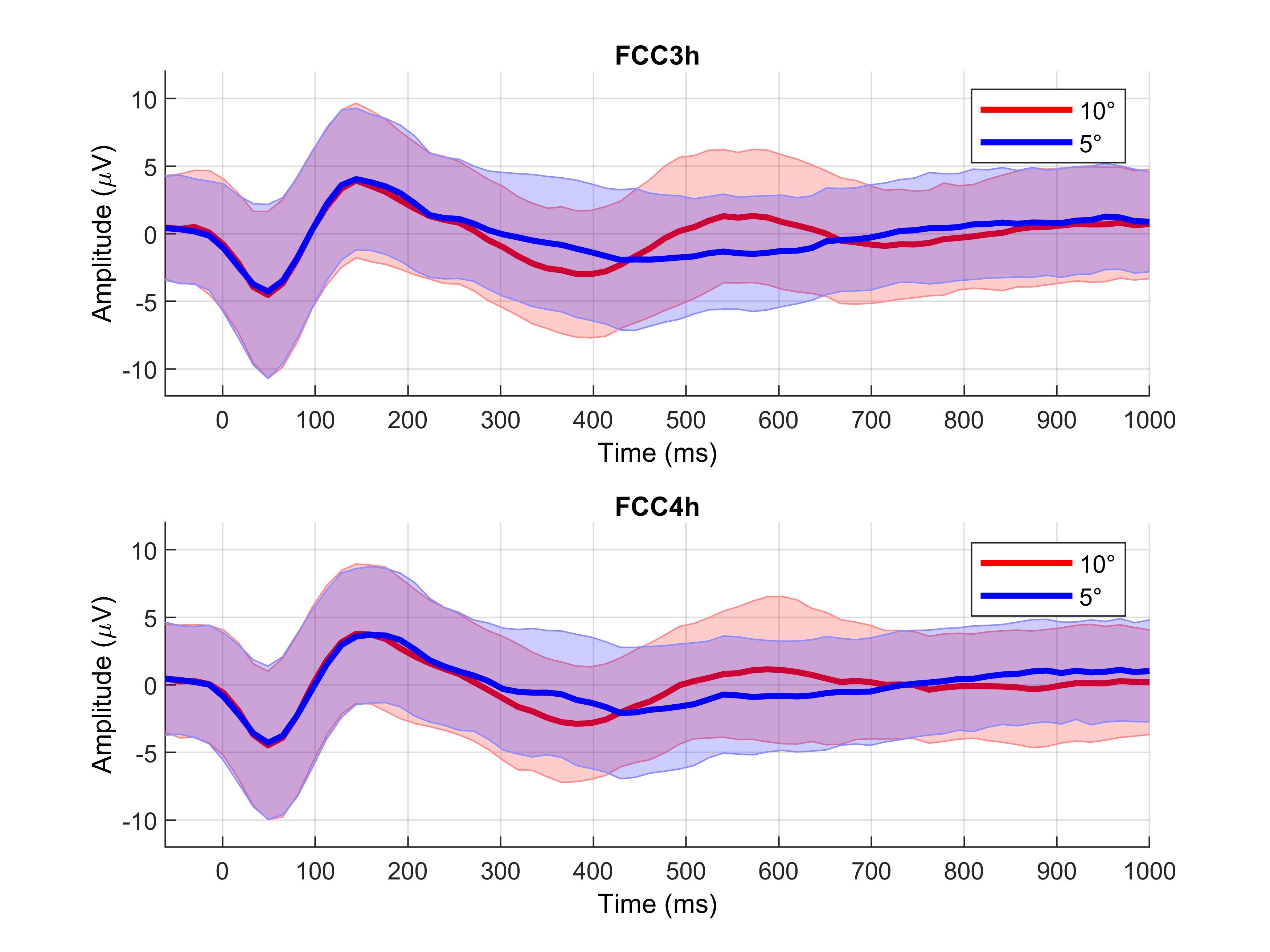


Figure 3. The first and second rows represents the PEPs of FCC3h and FCC4h respectively. Red and blue lines indicate the 10 and 5-degree trials.

Figure 4 illustrates the mean and STD of accelerometer data for each direction and angle separately.


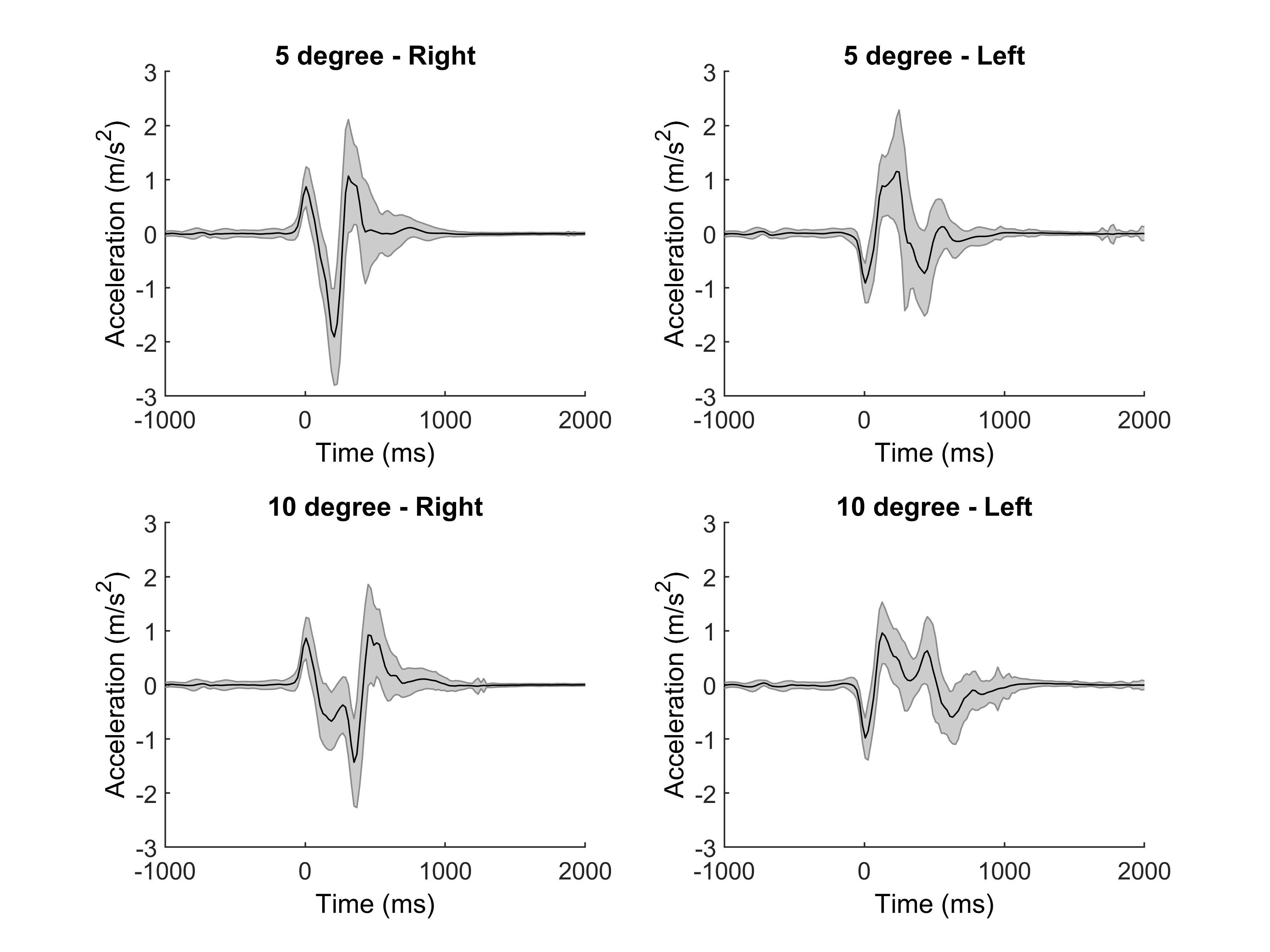


Figure 4. Abscissa of the accelerometer of Myo armband during perturbations. Acceleration data were plotted in the interval of [-1, 2] seconds with respect to onset of perturbation.

The obtained true positive rate (TPR) of each participant for binary and multi class scenarios are shown in Figure 5, the average TPR of 89.83% and 73.05% were achieved for two and four class conditions.


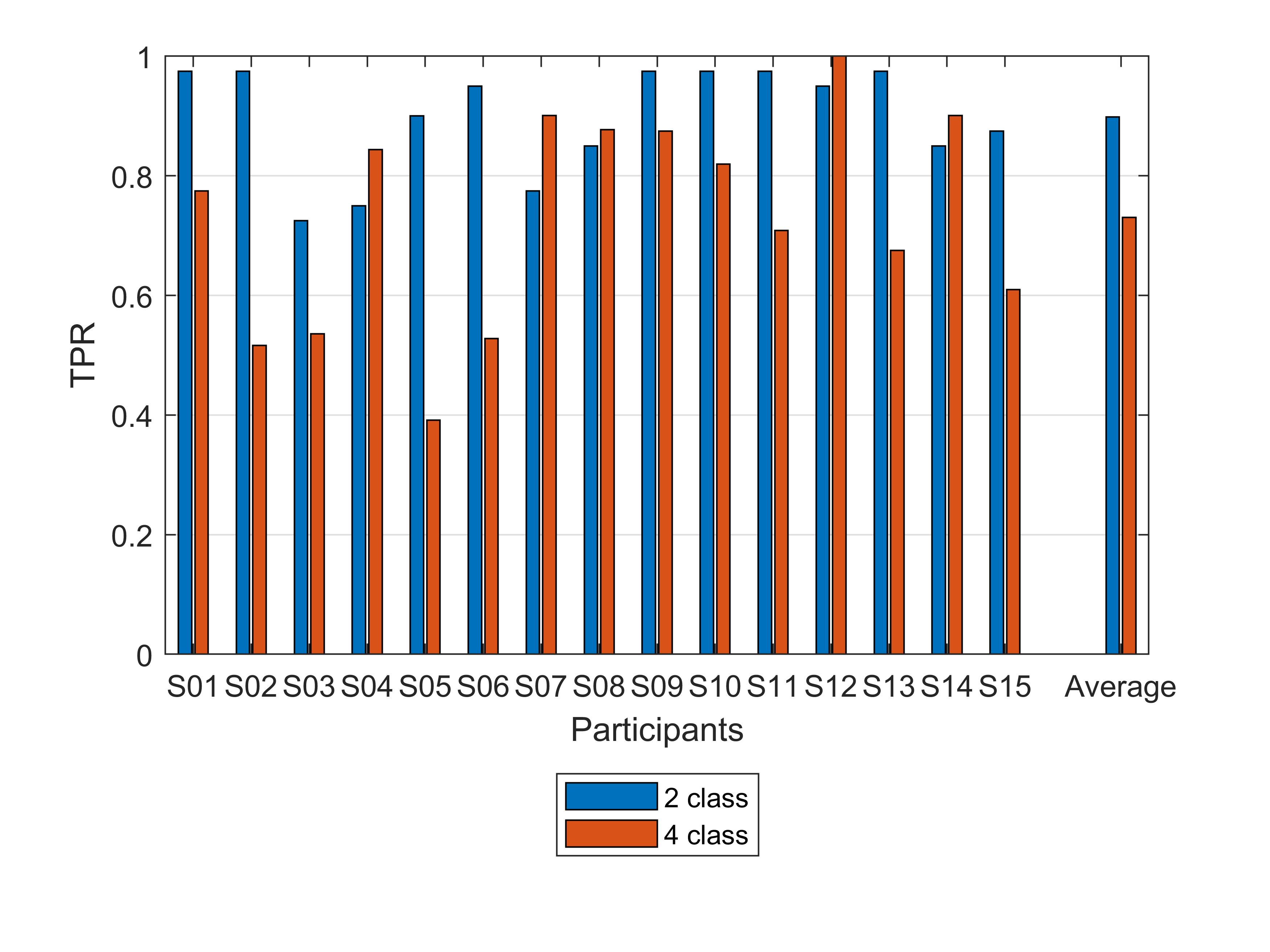


Figure 5. TPR of binary and multiclass classification of each participant were presented in blue and red colors respectively.
